# Targeting Rad51 as a strategy for the treatment of melanoma cells resistant to MAPK pathway inhibition

**DOI:** 10.1038/s41419-020-2702-y

**Published:** 2020-07-02

**Authors:** Elena Makino, Lisa Marie Fröhlich, Tobias Sinnberg, Corinna Kosnopfel, Birgit Sauer, Claus Garbe, Birgit Schittek

**Affiliations:** Division of Dermatooncology, Department of Dermatology, University of Tübingen, Tübingen, Germany

**Keywords:** Targeted therapies, Mechanisms of disease

## Abstract

Rad51 is an essential factor of the homologous recombination DNA repair pathway and therefore plays an important role in maintaining genomic stability. We show that *RAD51* and other homologous recombination repair genes are overexpressed in metastatic melanoma cell lines and in melanoma patient samples, which correlates with reduced survival of melanoma patients. In addition, Rad51 expression in melanoma cells was regulated on a transcriptional level by the MAPK signaling pathway with Elk1 as the main downstream transcriptional effector. Most strikingly, melanoma cells which developed resistance towards MAPK inhibitors could be efficiently targeted by Rad51 inhibitors similar to their sensitive counterparts, leading to DNA damage, G2/M arrest and apoptosis. Furthermore, the treatment of MAPK inhibitor resistant cells with Rad51 inhibitors enhances the susceptibility of these cells for MAPK inhibitor treatment in vitro and in vivo. These data indicate that Rad51 plays a critical role in the survival of metastatic melanoma cells and is a promising target for the therapy of melanoma irrespective of its MAPK inhibitor resistance status.

## Introduction

DNA damage repair pathways are of fundamental importance for genomic integrity and therefore play a critical role in cancer development and progression. Malignant melanoma is known to be a cancer with a high mutation burden caused by the accumulation of unrepaired UV-induced DNA damage^1,2^. However, some work has shown that DNA repair genes, which are particularly associated with homologous recombination repair (HRR), are overexpressed in melanomas with metastatic potential^3,4^. This suggests a benefit of maintaining a certain basal level of genetic stability during the process of metastasis^4,5^.

Rad51 is a central rate limiting protein of the HRR pathway and a critical protein for maintaining genomic stability. Rad51 is recruited to DNA regions in response to DNA double strand breaks or in case of replication fork stalling during and after DNA replication^6–8^. DNA double-strand break induction and recognition leads to nuclear accumulation of Rad51, which is detectable as discrete foci^9^. During these homology-directed repair processes, Rad51 stabilizes single-stranded DNA (ssDNA) by filament formation, searches for homologous DNA regions and mediates strand exchange^10,11^.

Rad51 overexpression frequently occurs in various cancers, including prostate cancer, breast and lung cancer as well as melanoma^12–15^ and is a negative prognostic marker for the survival of various cancer patients^16^. Increased expression of *RAD51* and other HRR-associated genes in tumor cells is supposed to enhance DNA repair and increase resistance to DNA damaging substances^17–19^.

Several mechanisms for the regulation of RAD51 level are already described. Among them, MAPK signaling pathway is often shown to mediate the transcription of *RAD51* mRNA^20–26^. MAPK inhibition in melanoma cells was recently shown to induce a HR deficient phenotype^22^.

Targeted therapy of patients with BRAF-mutated melanoma with either BRAF inhibitors or a combination of BRAF and MEK inhibitors has demonstrated a great success for the treatment of melanoma patients. However, the development of resistance remains the limiting factor for the long-term success of targeted therapy^27^. Therefore, it is essential to find new critical therapeutic targets in melanoma treatment to enable improved combination therapies. Within this work, we investigated the potential of Rad51 as therapeutic target in metastatic melanomas with or without acquired resistance to inhibitors of the MAPK pathway (MAPKi) as single agents or in combined treatments. We show that Rad51 may be a promising new target for the treatment of melanoma.

## Materials and methods

### Cell culture

The metastatic melanoma cell lines A375, SK-MEL19 and SK-MEL28 were purchased from ATCC. SbCl2 cell line was a gift of Dr. B. Giovanelli (Stehlin Foundation for Cancer Research, St. Joseph Hospital, Houston, TX). The other metastatic melanoma cell lines used here and the vemurafenib resistant patient derived xenograft (PDX) cells, WM4205-3, were kindly gifted by M. Herlyn and C. Krepler from the Wistar Institute (Philadelphia, USA). These cells were tested every 6 month to exclude mycoplasm contaminations. The cell lines SbCl2 and SK-MEL2 carry an *NRAS* mutation, whereas all other cell lines used here are *BRAF*-mutated cells. All cell lines were cultured in RPMI 1640 medium with 10% fetal bovine serum (Sigma Aldrich) and 1% penicillin and streptomycin (Thermo Fisher Scientific). The MAPKi resistant melanoma cells were generated as previously described^28^. The human primary melanocytes, fibroblasts and keratinocytes were isolated from human foreskin and cultured as previously described^29^. Stat3 overexpressing cells and corresponding control cells of the SK-MEL19 cell line were generated as described before^30^.

### Viability analysis

4-Methylumbelliferyl heptanoate (MUH; 100 µg/ml) was used for the analysis of cell viability as previously described^30^. The cells were treated for 3–5 days with vemurafenib (LC Laboratories, up to 20 µM), trametinib (Selleck Chemicals, up to 0.25 µM), B02 (Merck, up to 50µM^31^) or RI-1 (Sigma-Aldrich, up to 100 µM^32^) as indicated in the individual figures and figure legends. The equation: “log (inhibitor) vs. normalized response” was used for nonlinear regression analysis with the Graphpad Prism 6.0 software. The synergism analysis was performed using the following formula: *E*_exp_ = (*E*_drug1_ + *E*_drug2_) – (*E*_drug1_ × *E*_drug2_); *E*_exp_: expected effect of the combined treatment; *E*_drug_: Effect of a drug. The effect (E) represents the relative level of viability decline (0 < *E* < −1). The observed effect (*E*_obs_) is the relative level of viability decline after the respective combined drug treatment (synergistic effect: *E*_exp_ < *E*_obs;_ additive effect: *E*_exp_ = *E*_obs;_ antagonistic: *E*_exp_ > *E*_obs_).

### Cell cycle analysis

The distribution of the cells in the different cell cycle phases was analysed via propidium iodide (PI, Sigma-Aldrich, 50 µg/ml) staining and following FACS analysis as previously described^30^. The cells were treated with the indicated inhibitor concentrations for 5 days.

### Spheroid assay

Hanging drops of melanoma cells (250 cells per 25 µl drop) were cultured for 10 days in normal growth medium to allow spheroid formation. The melanoma spheroids were collected and 5–10 spheroids per 12-well plate cavity were embedded in collagen I (Sigma-Aldrich, 1.2 mg/ml).

The spheroids were treated in triplicates with the indicated concentration of MAPK inhibitors (vemurafenib, trametinib) and/or RAD51 inhibitors (B02, RI-1) on day 0 and day 3. Light microscopic images were taken on days 0, 3 and 5. Image J software was used to quantify the spheroid size. For this purpose, the areas of 10 spheroids per treatment group were determined in one experiment and the data of three independent experiments were summarized. Calcein AM (Merck, 2 µM) and PI (Merck, 8 µg/ml) were used for alive/dead staining and the images were taken using an fluorescence microscope (Zeiss) and VisiView® software (Visitron Synstems GmbH).

### Colony formation assay

In all, 1 × 10^3^ melanoma cells were seeded into each cavity of a 12-well plate. In all, 24 h later, these cells were treated with the indicated concentrations of MAPK inhibitors (vemurafenib, trametinb) and/or RAD51 inhibitors (B02, RI-1) for 7 to 12 days. The inhibitor-containing medium was changed every 3 days. The cells were fixed in 4% formalin and stained with 3% crystal violet solution (Sigma-Aldrich) in 80% methanol for 2 h.

### RAD51 promotor-reporter assay

Secrete-Pair ™ Dual Luminescence Assay Kit with GLuc-ON Promoter Reporter Clone (HPRM36743-PG04) encoding the RAD51 promoter (Gene Accession: NM_001164270; Genome location: chr15 + :40693712-40695392 with transcription start site = 40695129 (assembly version: hg38); GeneCopoeia^TM^) were used to analyze the RAD51 promoter activity via the Gaussia luciferase normalized to the secreted alkaline phosphatase signals according to the manufacturer’s protocol. The reporter plasmid (2 µg/well) and siRNA against Elk1 (20 nM) were cotransfected with the transfection reagent GenaxxoFect (Genaxxon bioscience) into the melanoma cells in six-well-plates (4 × 10^5^ cells/well). In all, 24 h after transfection, these melanoma cells were re-seeded in 96 well-plates (10^4^ cells/well). After attachment of the cells they were treated with DMSO, 2 µM vemurafenib or 20 nM trametinib. The control cells remained without any treatment. The supernatant was collected 72 h after transfection. The Gaussia luciferase activity or the secreted alkaline phosphatase activity (SEAP) was measured in 5 µl of the 300 µl supernatant.

### Chromatin immunoprecipitation

Chromatin immunoprecipitation (ChIP) was performed using SimpleChiP^®^ Plus Enzymatic Chromatin IP Kit (Cell Signaling Technology) according to the manufacturer’s protocol. In all, 4 × 10^6^ cells were used for chromatin purification and subsequent respective immunoprecipitation with either Histone H3 (D2B12) XP^®^ Rabbit mAb (CST, positive control) or ELK1 antibody (ab32106, Abcam)). The enrichment of the DNA sequences was analyzed by RT-qPCR using GoTag®qPCR Master Mix (Promega) in the LightCycler^®^ 96 system (Roche). The SimpleChIP^®^ Human RPL30 Exon 3 Primers (#7014, CST) were used as positive control. The following primer pair was used for the detection of the respective Rad51 promotor sequence: forward: 5′-TCTTCTCGAGCTTCCTCAGC-3′, reverse: 5′-AGCGCTCTTGTGGTTTGTTT-3′). The detected signals were normalized using the percent input method (%input = 100 × $2^{({\rm{Ct}}_{100\% {\rm{input}}} -{\rm{Ct}}_{\rm{IP}})}$; Ct_100%input_ = Ct_input_ − log_2_ (dilution factor)).

### RNA isolation and RT-qPCR

The RNA isolation and RT-qPCR analysis were performed as previously described^28^. A 96 block Lightcycler (Roche) was used. The primer sequences are indicated in the supplementary table 1.

### Immunoblot analysis

The analysis was performed as described before^30^. The following primary antibodies were used: anti-Rad51 (1:10000, Abcam, ab133534), anti-p-ERK (1:1000, CST, #4370), anti-ERK (1:1000, CST), anti-Actin (1:1000, CST, #4696), anti-pH2AX (Ser139) (1:500, CST, #9718), anti-p-Elk-1 (1:250, Santa Cruz Biotechnology, B-4, sc-8406), anti-Elk-1 (1:250, Santa Cruz Biotechnology, E-5, sc-365876), anti-Stat3 (1:1000, CST, #9139), anti-cMyc (1:1000, CST, #5605), anti-p-Stat3(Ser727) (1:250, Santa Cruz, sc-8001-R), anti-Caspase-3 (1:1000, CST, #9668), anti-cleaved Caspase-3 (1:1000, CST, #9664).

### Analysis of apoptosis, DNA damage, and cell proliferation

The Apoptosis, DNA Damage and Cell Proliferation Kit (BD Biosciences) was used for the staining of pH2AX and cleaved PARP as well as for the BrdU incorporation assay. The analysis was performed according to the manufacturer´s protocol by FACS analysis (LSRII and FACS DIVA software).

### PCR array of DNA damage repair genes

RT² Profiler™ PCR Array Human DNA Repair (PAHS-042, Qiagen) was used for the analysis of DNA repair gene expression. The assay and data analysis were performed according to the manufacturer’s instructions. Gene expression in the A375 cell line treated with DMSO (0.02%, 24 h) or vemurafenib (2 µM, 24 h) was compared (*n* = 3).

### HRR reporter analysis

In all, 2.5 × 10^5^ cells per six-well cavity were seeded and incubated for 24 h. These cells were transiently transfected with pDRGFP plasmid (Addgene, 1 µg/well) and pCBASceI plasmid (Addgene, 1 µg/well) using Lipofectamine 3000 (Thermo Fisher Scientific) according to the manufactures protocol to determine the capacity of HRR^33^. In all, 24 h later, the cells were treated with B02 or RI-1 for 3 h, respectively. The proportion of GFP positive cells indicating the HRR capable cells was determined via FACS analysis (LSRII and FACS DIVA software). The analysis was performed using FlowJo software.

### Immunofluorescence staining

In all, 2.5 × 10^4^ cells were seeded to each cavity of a four-chamber slide and the treatments with MAPKi, RAD51i or cisplatin (Hexal) started 24 h later as indicated in the figure legends. The cells were fixed by 4% Formalin for 15 min. and were stained using the primary antibodies, anti-pH2AX (Ser139) (1:500, Merck, JBW301) and anti-Rad51 (1:500, Abcam, ab133534). The nuclei were stained by DAPI (Invitrogen, NucBlueTM fixed cell stain ReadyProbesTM reagent) or Yo-PRO (Thermo Fisher Scientific). The following secondary antibodies were used: AlexaFluor 647-conjugated Donkey Anti-Mouse IgG or Cy-3 conjugated Donkey Anti-Mouse IgG (1:250, Dianova) for the detection of pH2AX and Fluorescein (FITC)-conjugated Donkey Anti-Rabbit IgG (1:200, Jackson Immuno Research Laboratories) for the detection of Rad51. The pictures were taken by fluorescence microscope (Zeiss/VisiView software and Nikkon/Nikkon-Capture NX-D software). Rad51 and pH2AX foci of 50 to 100 cells per treatment group were counted for quantification.

### Small interfering RNA (siRNA) transfection

siRNAs (siGENOME human, Dharmacon) were transfected with Lipofectamine RNAimax (Thermo Fisher Scientific) according to the manufactur´s protocol as described before^28^. The transfected cells were used for MUH viability analysis 24 h after the start of transfection. The immunoblot samples were collected after 48 h.

### Immunohistochemical staining

Formalin-fixed paraffin-embedded (FFPE) tissue sections of metastatic melanoma samples were de-paraffinized and prepared as previously described^30^. Samples from 25 metastatic melanoma patients were used. Rad51 (1:200, Abcam, ab133534) staining with Fast Red Substrate (Thermo Scientific Lab Vision Liquid Fast-Red Substrate System) and additional hematoxylin counterstaining is shown. This analysis was approved by the Ethics Commission Tübingen, Germany (approval number 800/2016B02).

### Xenograft mice experiment

In all, 1.0 × 10^6^ A375R melanoma cells were subcutaneously injected into the flanks of NSG mice. The mice were randomized into four groups using Microsoft Excel, taking gender into account (nine mice per treatment group) after the formation of palpable tumors of 25 mm^3^. The mice received an intraperitoneal injection of B02 (50 mg/kg) or normal saline (NS) every 3 days, for a total of four times. B02 was dissolved in Cremophor, DMSO and NS in the ratio of 1∶1∶3. In addition, the mice were fed with vemurafenib (LC Laboratories, 417 mg/kg in feed) or standard food for 15 days (ssniff Spezialdiäten GmbH). The treatments were performed in unblinded way. They were euthanized if the tumors reached a size of more than 1000 mm^3^ or if the tumors were ulcerated. Animals were excluded if no palpable tumors had formed 3 weeks after tumor cell injection. The tumor volume was analyzed by length and thickness measurement and using the formula “V_tumor_ = 0.5 × width^2^ × length”. Female (*n* = 13) and male (*n* = 13) mice aged between 15 and 20 weeks at the beginning of the experiments were used. The sample size of 9 animals per group was estimated based on preliminary data, in order to detect difference using the unpaired two-sample Students’s *t*-test at a power of 90%. The mice experiments were performed in compliance with the requirements of the German Animal Welfare Act and approved by the regional council (Regierungspräsidium Tübingen, HT1-18).

### Database and statistical analysis

The statistical analysis was performed using the Graphpad Prism 6.0 software and Microsoft Excel by unpaired Student’s *t*-test or Multiple *t*-test according to Holm Sidak method as indicated in the respective figure legends. The statistically significant data (*p* < 0.05) were marked with asterisks (* for *p* < 0.05, ** for *p* < 0.01 and *** for *p* < 0.001) in the graphs. Significance analysis of the survival curve in the xenograft experiment was performed using the Kaplan–Meier method and the subsequent Mantel-Cox test. The Kaplan–Meier curves of melanoma patient data were generated using RNAseq data from 375 cutaneous primary and/or metastatic melanomas of the TCGA-SKCM project^34^ using R2 genomics analysis and visualization platform (https://r2.amc.nl). The expression data of the patient tumors were separated into high (*n* = 174) and low (*n* = 201) expression groups and a survival cut-off at 10-year time point was chosen.

## Results

### Melanoma cells exhibit a high expression of Rad51 resulting in increased DNA damage repair

**Fig. 1:**
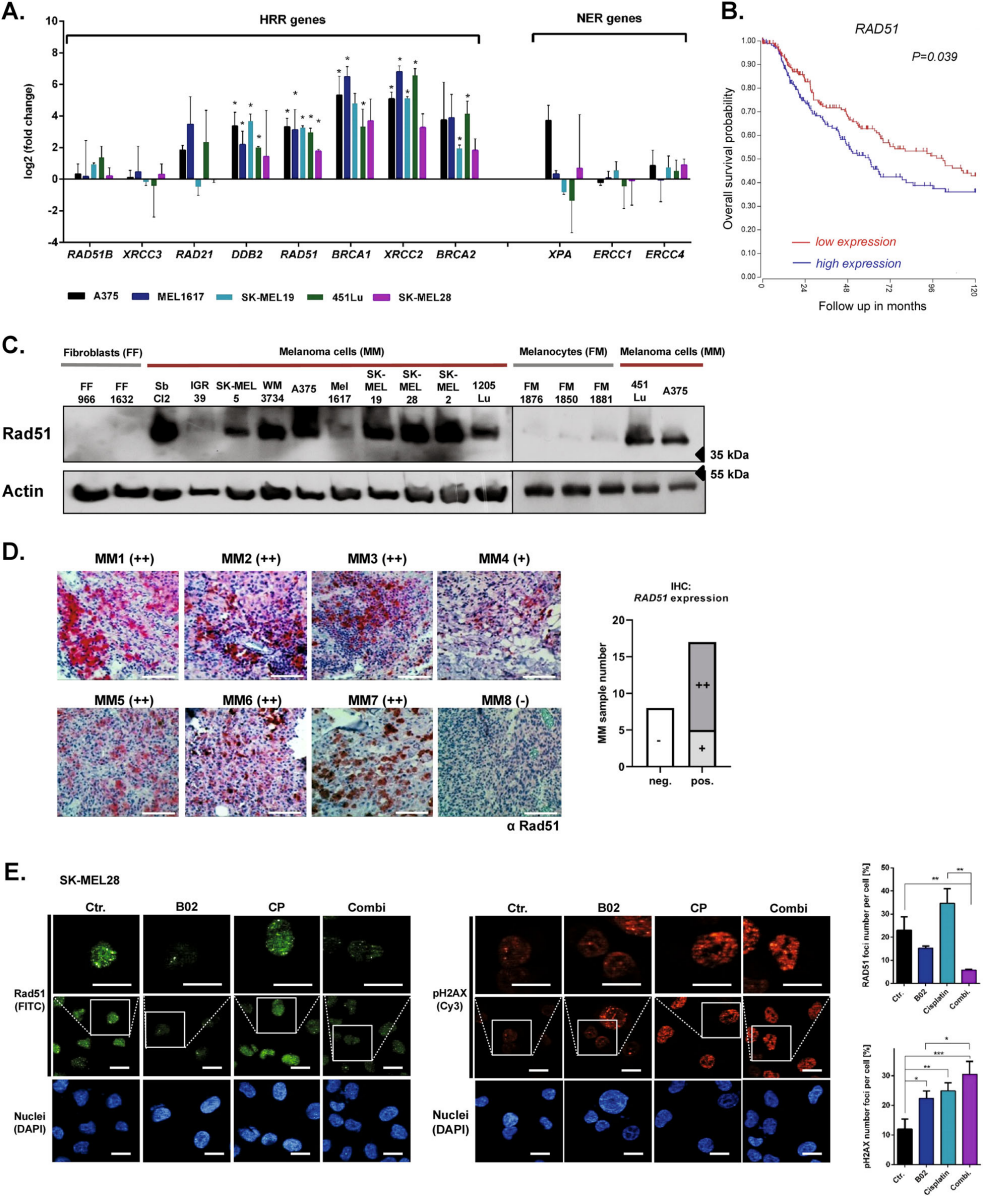
Melanoma cells exhibit a high expression of Rad51 resulting in increased DNA damage repair. **a** The basal mRNA level of different homologous recombination repair (HRR) and nucleotide excision repair (NER) genes in melanoma cell lines, normalized to respective actin expression is shown relative to expression in melanocytes (RT-qPCR, *n* = 3, mean ± SEM, unpaired Student’s *t*-test *p < 0.05). **b** Overall survival of melanoma patients depending on *RAD51* expression (high expression: blue line, *n* = 174, low expression: red line, *n* = 201). Cutaneous melanoma (SKCM) patient data from the TCGA database were used. **c** The basal protein level of Rad51 was determined in melanocytes (FM), fibroblasts (FF) or various melanoma cell lines (MM) by immunoblot analysis. **d** Exemplary pictures of immunohistochemical staining (IHC) for Rad51 in metastatic melanoma patient samples (MM) (Fast Red substrate) with hematoxylin counterstaining (scale bar: 200 μm, left pictures). The Rad51 expression level was distinguished between high expression (++, pos.), intermediate expression (+, pos.) as well as low/no expression (−, neg.) (right graph). **e** Immunofluorescence staining for Rad51 (FITC, green), pH2AX (Cy3; red), and nuclei (DAPI; blue) after treatment with B02 (B02, 10 µM, 1 h) and/or subsequent addition of cisplatin (CP 20 µM, 6 h). Control cells (Ctr.) were treated with DMSO (0.05%) (left pictures). The Rad51 and pH2AX foci number per cell were quantified (right graph). Scale bars: 20 µm (*n* = 3, mean ± SEM, unpaired Student’s *t*-test, *:for *p* < 0.05, ** for *p* < 0.01).

To gain an impression of the importance of DNA repair processes in melanoma cells, we first compared gene expression associated with HRR and nucleotide excision repair (NER) in metastatic melanoma cell lines and primary human melanocytes. We found that several HRR-associated genes, including *DDB2*, *RAD51*, *BRCA1*, *XRCC2* and *BRCA2*, are overexpressed in melanoma cells compared to primary melanocyte cultures, while the expression of the NER-associated genes *XPA*, *ERCC1* and *ERCC4* showed no clear differences (Fig. 1a).

Next, we analyzed whether the expression level of HRR-associated genes affects overall survival of melanoma patients using the TCGA database. Among the different genes of the HRR pathway, especially the expression level of the *RAD51* gene has a negative influence on the survival of melanoma patients (Fig. 1b). In contrast, we found no significant differences in patient survival in the two groups expressing higher or lower levels of the other HRR-associated genes (Supplementary Fig. 1A).

Therefore, we focused on Rad51 expression and confirmed that also Rad51 protein was strongly expressed in most human melanoma cell lines, in contrast to primary human melanocytes (FM) and primary human fibroblasts (FF), which have either no or very low levels of Rad51 (Fig. 1c). Similar to the observations in melanoma cell lines, we show high Rad51 expression in metastatic melanoma tumor samples from 17 out of 25 patients by immunohistochemical staining (Fig. 1d, Supplementary Fig. 1B).

Since increased HRR genes expression and in particular the expression of *RAD51* mediate an upregulation of HRR capacity in cancer cells, we have analyzed whether Rad51 protects melanoma cells from DNA damage by increasing HRR capacity. Therefore, we have analyzed the formation of nuclear Rad51 foci and pH2AX foci after genotoxic stress via cisplatin treatment in melanoma cells with or without prior Rad51 inhibition. Indeed, Rad51 foci induction by cisplatin treatment was blocked through previous treatment with Rad51 inhibitor (Rad51i) B02 (Fig. 1e). We also show that treatment with cisplatin leads to an increased pH2AX foci number, indicating the accumulation of DNA double-strand breaks, which was further enhanced by previous treatment with Rad51i (Fig. 1e).

These data suggest a critical role of Rad51 overexpression for effective DNA damage repair and thus for the survival of metastatic melanoma cells.

### *RAD51* gene expression is regulated by the MAPK signaling pathway in melanoma cells via Elk1

**Fig. 2:**
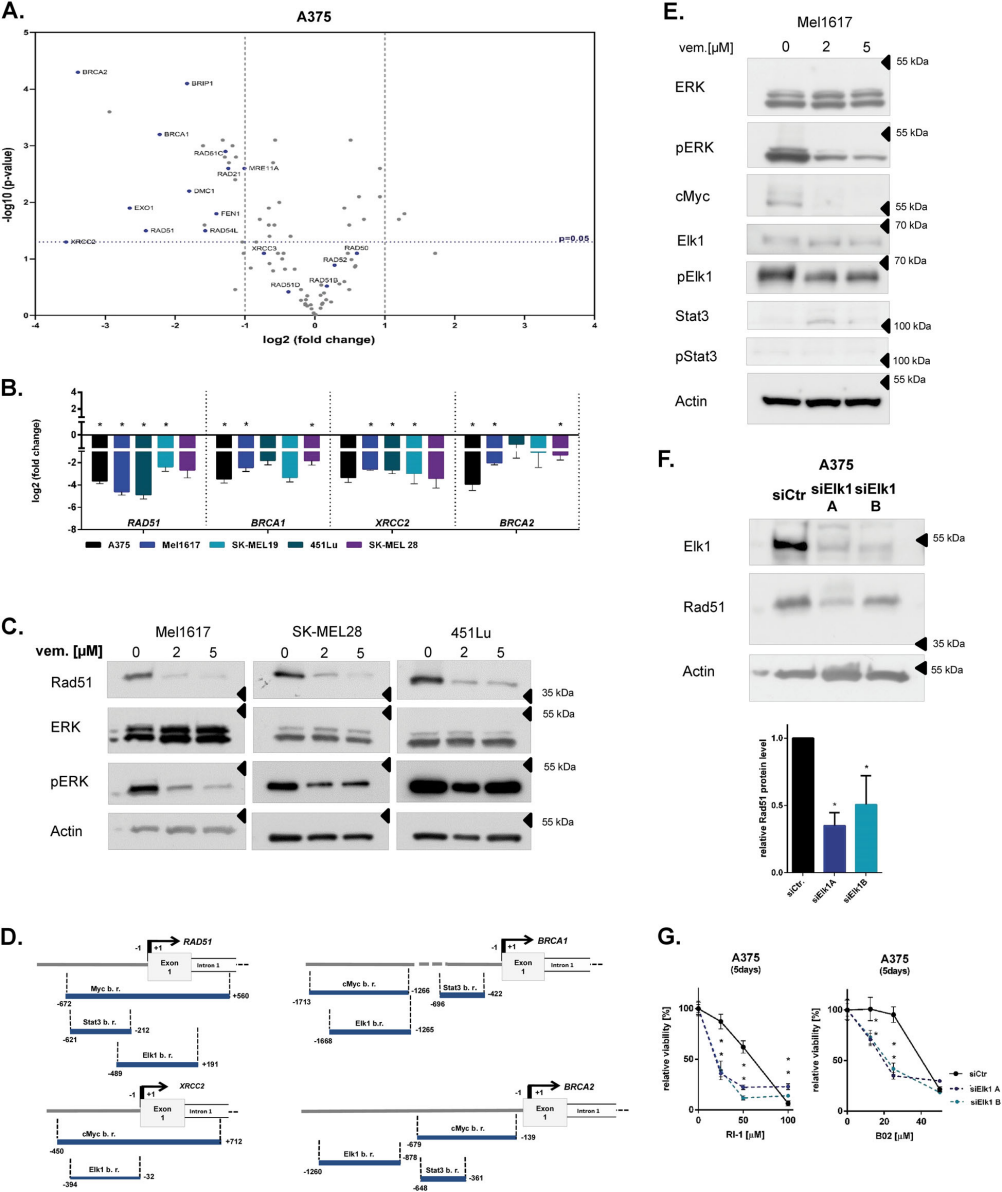
*RAD51* gene expression is regulated by the MAPK signaling pathway in melanoma cells via Elk1. **a** The influence of vemurafenib treatment (2 µM, 24 h) on the expression of 84 different DNA repair genes in the A375 cell line was analyzed by PCR array. DMSO (0.02%, 24 h) treated cell samples served as control. log2 fold changes in the expression of the corresponding genes between the vemurafenib treated group and the control group and log10 p-values of the respective gene expression changes (unpaired Student’s *t*-test) are shown in the volcano plot (*n* = 3 per group). The data of the HRR genes are marked in blue and named. The data of other DNA repair genes are shown as gray dots. **b** The log2 of fold change in the expression of HRR genes between the vemurafenib (2 µM, 24 h) treated samples and DMSO (0.02%, 24 h) treated control samples from five melanoma cell lines is plotted (RT-qPCR, *n* = 3 in triplicates, mean ± SEM, unpaired Student’s *t*-test *: *p* < 0.05). **c** Immunoblot analysis of vemurafenib (vem., 2 or 5 µM, 24 h) treated or untreated (0) melanoma cell line samples. **d** The binding sites of the critical transcription factors in the indicated HRR gene promoter regions were determined via the ENCODE website (b.r.: binding region). **e** Immunoblot analysis of vemurafenib (vem., 2 or 5 µM, 24 h) treated or untreated (0) Mel1617 cell line samples. **F**. Elk1 knockdown was induced by respective siRNA transfection (siElk1A and siElk1B) in A375 cells and its effect on Rad51 expression was analyzed by immunoblot analysis (48 h after transfection). Samples of non-coding siRNA transfected cells were used as control (siCtr.). The Rad51 protein level in the respective samples were quantified and normalized to the correspondent actin level (lower graph, *n* = 3, mean ± SD, Unpaired Student’s *t*-test, *: *p* < 0.05) **g** Elk1 knockdown cells (siElk1A and siElk1B) or Control cells (siCtr.) described in F. were treated for 5 days with different concentrations of Rad51is (B02 or RI-1) (viability analysis in quituplicates, mean ± SD, Multiple *t*-test according to Holm Sidak method, * for *p* < 0.05).

Next, we asked whether the high expression of HRR genes is influenced by the MAPK signaling pathway in melanoma cells. Gene expression array analysis of 84 DNA damage repair genes showed that twelve out of seventeen analysed HRR genes are downregulated via MAPK signaling pathway inhibition by the BRAF inhibitor (BRAFi) vemurafenib (Fig. 2a, Supplementary Fig. 2A).

We have confirmed the regulation of four exemplary genes, namely *RAD51*, *BRCA1, BRCA2*, and *XRCC2* by vemurafenib treatment in further BRAF-mutated melanoma cell lines using RT-qPCR analysis at the mRNA level (Fig. 2b) and the regulation of Rad51 at protein level using immunoblot analysis (Fig. 2c). This was not due to the effect of MAPK inhibitors (MAPKi) on the cell cycle phase distribution (Supplementary Fig. 2B).

To search for the specific transcription factors of the MAPK pathway that regulate HRR-associated gene expression, we compared known MAPK pathway effectors with described transcription factors binding sites in the gene promoter regions of the twelve HRR-associated genes, which are downregulated by vemurafenib treatment. We found three critical candidates, namely Stat3, Elk1 and cMyc (Fig. 2d, Supplementary Fig. 2C). Indeed, vemurafenib treatment of melanoma cells led to a reduction in c-Myc and Elk1 expression and Elk1 phosphorylation, while Stat3 expression and phosphorylation were not affected (Fig. 2e).

To test whether cMyc, Stat3 and/or Elk1 regulate *RAD51* expression, we downregulated each transcription factor by specific siRNA and analyzed the effect on Rad51 level. The siRNA-mediated Elk1 knockdown achieved a significant reduction in Rad51 protein level (Fig. 2f), while Stat3 and cMyc knockdown as well as overexpression of Stat3 did not produce clear changes in Rad51 expression (Supplementary Fig. 2D, E). Furthermore, the binding of Elk1 to the *RAD51* promoter was confirmed by a ChIP analysis as well as by a reporter analysis detecting the activity of the *RAD51* promoter (Supplementary Fig. 2F, G). Additional inhibition of the MAPK pathway reduced reporter activity to 70% and Elk1 knockdown reduced it to 50%, confirming the role of Elk1 as one of the major transcription factors for *RAD51* gene regulation in melanoma cells (Supplementary Fig. 2G). On the functional level, the Elk1 knockdown increased the sensitivity of melanoma cells to the two Rad51i used, B02 and RI-1, most likely due to the double reduction of Rad51 function (Fig. 2g), on the one hand by Rad51i and on the other hand by the Rad51 deregulation mediated by Elk1.

These data suggest that Elk1 is an important transcription factor regulated by MAPK signaling pathway which regulates downstream HRR gene expression like *RAD51*.

### MAPKi sensitive and resistant metastatic melanoma cells are susceptible to Rad51 inhibition

**Fig. 3:**
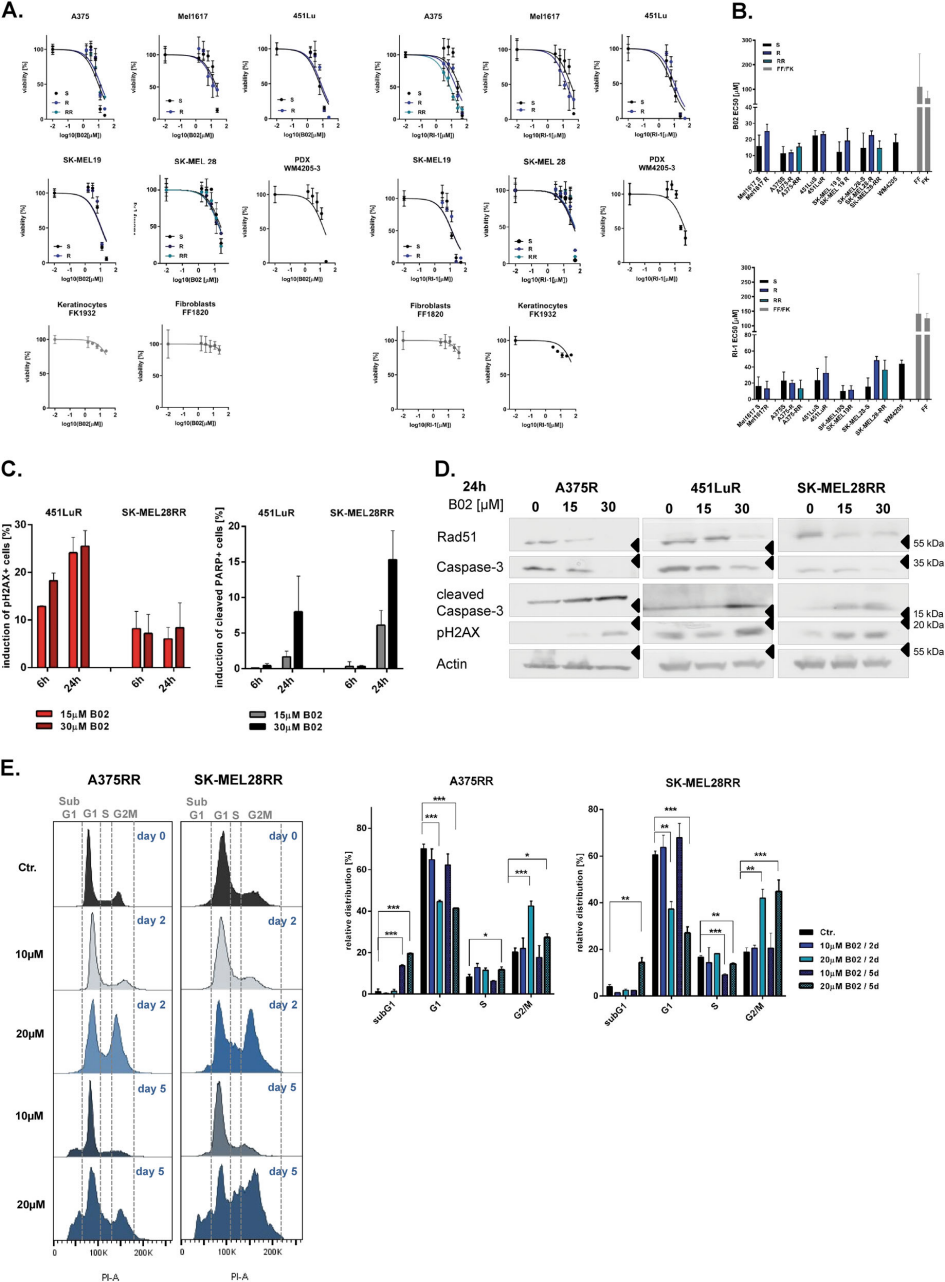
MAPKi sensitive and resistant metastatic melanoma cells are susceptible to Rad51 inhibition. **a** Vemurafenib sensitive (S), resistant (R) and vemurafenib/trametinib double resistant (RR) melanoma cell lines as well as fibroblasts (FF) and keratinocytes (FK) were treated with different concentrations of Rad51i (B02 or RI-1) for 5 days (viability analysis, exemplary data of one experiment, mean ± SD, PDX: patient derived xenograft cells). **b** EC50 values of Rad51 is in the corresponding cell lines were calculated using the data of the viability analysis described in **a** (*n* = 3, mean ± SEM). **c** The increase of pH2AX-positive (left graph) and cleaved PARP-positive cell population (right graph) after B02 treatment (15 or 30 µM) for 6 h or 24 h was detected by FACS staining (*n* = 2, mean ± SEM). **d** Immunoblot analysis of melanoma cells treated with B02 (15 or 30 µM) for 24 h and respective untreated control cells (0). **e** Cell cycle analysis was performed after treatment with B02 (10 or 20 μM) for 2 or 5 days. Exemplary data of one experiment is displayed graphically (left graph). The relative distribution in the different phases of the cell cycle is quantified (right graph, *n* = 3, mean ± SD, Multiple *t*-tests using the Holm-Sidak method, * for *p* < 0.05, ** for *p* < 0.01 and *** for *p* < 0.001).

Due to the finding of a high Rad51 level in melanoma cells, we have asked for its potential as a target protein in melanoma therapy. We analyzed the sensitivity to two small molecule inhibitors of Rad51 B02 and RI-1 in treatment-naïve metastatic melanoma cell lines (S) as well as particularly in the respective BRAFi (vemurafenib) resistant (R) and BRAFi and MEKi (vemurafenib and trametinib) double resistant (RR) melanoma cell lines. One patient-derived xenograft melanoma cells (PDX) isolate from a melanoma patient who developed acquired resistance to the BRAFi vemurafenib was used as well (Supplementary Fig. 3A, Fig. 3a, b). The viability analysis data show that inhibition of Rad51 strongly reduces the viability of all melanoma cells analysed, while the viability of other skin cells, including fibroblasts and keratinocytes, remained largely unaffected (Fig. 3a, b). Interestingly, R and RR cells retain their sensitivity to Rad51 inhibition because their EC50 values for Rad51 inhibition were not significantly different from those of the corresponding S cells (Fig. 3b). In accordance with these data, we do not see any differences between the Rad51 level of R or RR cells and the respective parental cell line (Supplementary Fig. 3B).

Since Rad51 plays a crucial role in repairing DNA double strand breaks and restarting DNA replication when the replication fork stops, we have analyzed the effects of Rad51i treatment on these mechanisms. We found that Rad51i treatment initially leads to an accumulation of DNA damage, which is reflected in an increased number of pH2AX foci detected by both FACS analysis (Fig. 3c) and immunoblot analysis (Fig. 3d). In addition, we confirmed the specific inhibition of HRR in reporter melanoma cells by the respective Rad51i treatment (Supplementary Fig. 3C).

The cell cycle analysis shows that the melanoma cells treated with Rad51i initially remain in the G2/M phase after two days of treatment (Fig. 3e). In accordance with these data, we found that Rad51 inhibition after this time also reduces the capacity of DNA replication (Supplementary Fig. 3D). Treatment for another three days finally leads to apoptosis induction, measured by an elevated sub-G1 fraction (Fig. 3e). The apoptosis induction by Rad51 inhibition was additionally confirmed by an increased amount of cleaved PARP and cleaved Caspase-3 as demonstrated by FACS staining (Fig. 3c) or immunoblot analysis (Fig. 3d).

These data show that melanoma cells are sensitive to Rad51 inhibition and remain responsive even after MAPKi resistance has been acquired.

### Co-inhibition of the MAPK pathway and Rad51 reduces viability of MAPKi resistant melanoma cells in a synergistic manner

Next, we asked whether MAPKi resistant melanoma cells respond to the combined inhibition of MAPK pathway and Rad51.

Treatment of A375 S and R cells with increasing concentrations of vemurafenib downregulated Rad51 protein level in the cytoplasm and nucleus in a dose-dependent manner, while higher vemurafenib concentrations are required in R cells (Supplementary Fig. 4A). However, dual inhibitor treatment with lower concentrations of MAPKi and Rad51i also reduced Rad51 levels in the R cell line (Supplementary Fig. 4B). This effect can also be seen in immunofluorescence analysis (Supplementary Fig. 4C). These data suggest a synergistic reduction of Rad51 expression by dual inhibition using sub-effective single inhibitor concentrations.

**Fig. 4:**
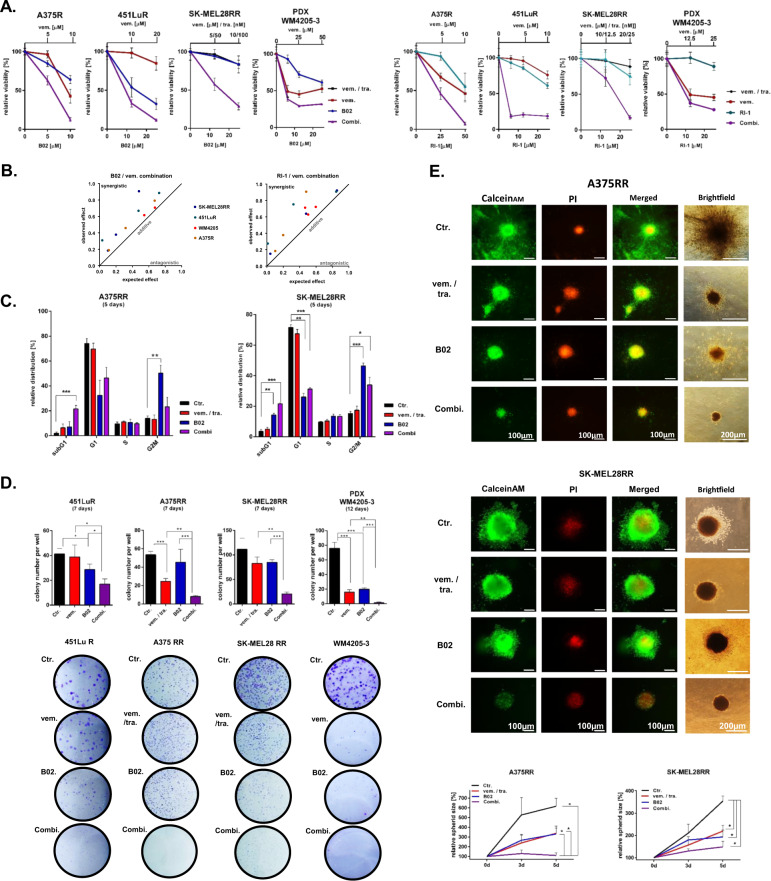
Co-inhibition of the MAPK pathway and Rad51 reduces viability of MAPKi resistant melanoma cells in a synergistic manner. **a** R and RR melanoma cell lines were treated with indicated concentrations of vemurafenib (vem.), trametinib (tra.), Rad51i (B02 or RI-1) or a combination of respective inhibitors for 5 days (viability analysis, exemplary data of one experiment, mean ± SD). **b** The data from **a** were used for synergism analysis. **c** Cell cycle analysis was performed after treatment with vemurafenib (vem., 5 µM), trametinib (tra., 50 nM), B02 (10 µM) or a combination of the corresponding inhibitors for 5 days (PI staining). DMSO-treated cells (0.02%) served as control (Ctr.) (*n* = 3, mean ± SEM, Multiple *t*-tests using the Holm-Sidak method). **d** Colony formation was analyzed after treatment with vemurafenib (vem., 5 µM), additional treatment with trametinib (tra., 25 nM), B02 (10 µM) or the indicated combination of these inhibitors for 7 or 12 days. Exemplary images of the colonies are shown (lower graph). Colony number per well of a twelve well plate was counted and analyzed (*n* = 3, mean ± SD, unpaired Student’s *t*-test). **e** Melanoma spheroids were treated with DMSO (Ctr., 0.02%), vemurafenib (vem. 5 µM), trametinib (tra. 100 nM), B02 (15 µM) or a combination of these inhibitors in collagen for 5 days (upper graph, live (green) / dead (red) staining). The total sizes of 5–10 spheroids per treatment group were quantified (lower graph, *n* = 3, mean ± SEM, Multiple *t*-test according to Holm Sidak method). **c–e** All significant differences are marked as asterisks with * for *p* < 0.05, ** for *p* < 0.01 and *** for *p* < 0.001.

We further investigated the functional effect of dual inhibitor treatments. The combined inhibition of the MAPK pathway and Rad51 increased the effect on the viability of R, RR, and PDX melanoma cells (Fig. 4a) compared to the effects of single agent treatments. Additional analyses of these data show that combination treatment reduces the viability of melanoma cells in a synergistic manner (Fig. 4b), which can be explained by the resulting co-inhibition of HRR by these inhibitors.

In addition, co-treatment also leads to increased accumulation of DNA damage (Supplementary Fig. 4D), increased apoptosis induction (Fig. 4c, Supplementary Fig. 4E), and reduced colony formation capacity (Fig. 4d, Supplementary Fig. 4F) compared to single inhibitor treatment. We also used a 3D spheroid model to investigate this issue. We show that, in particular, the combined inhibitor treatment for five days reduces the size and viability of melanoma cell spheroids (Fig. 4e, Supplementary Fig. 4G). Similar to the effect of Rad51 inhibition, the siRNA-mediated Rad51 knockdown sensitized the R and RR melanoma cells for MAPKi treatment (Supplementary Fig. 4H).

### MAPKi resistant melanomas can be effectively treated with a combination of MAPKi and Rad51i in vivo

**Fig. 5:**
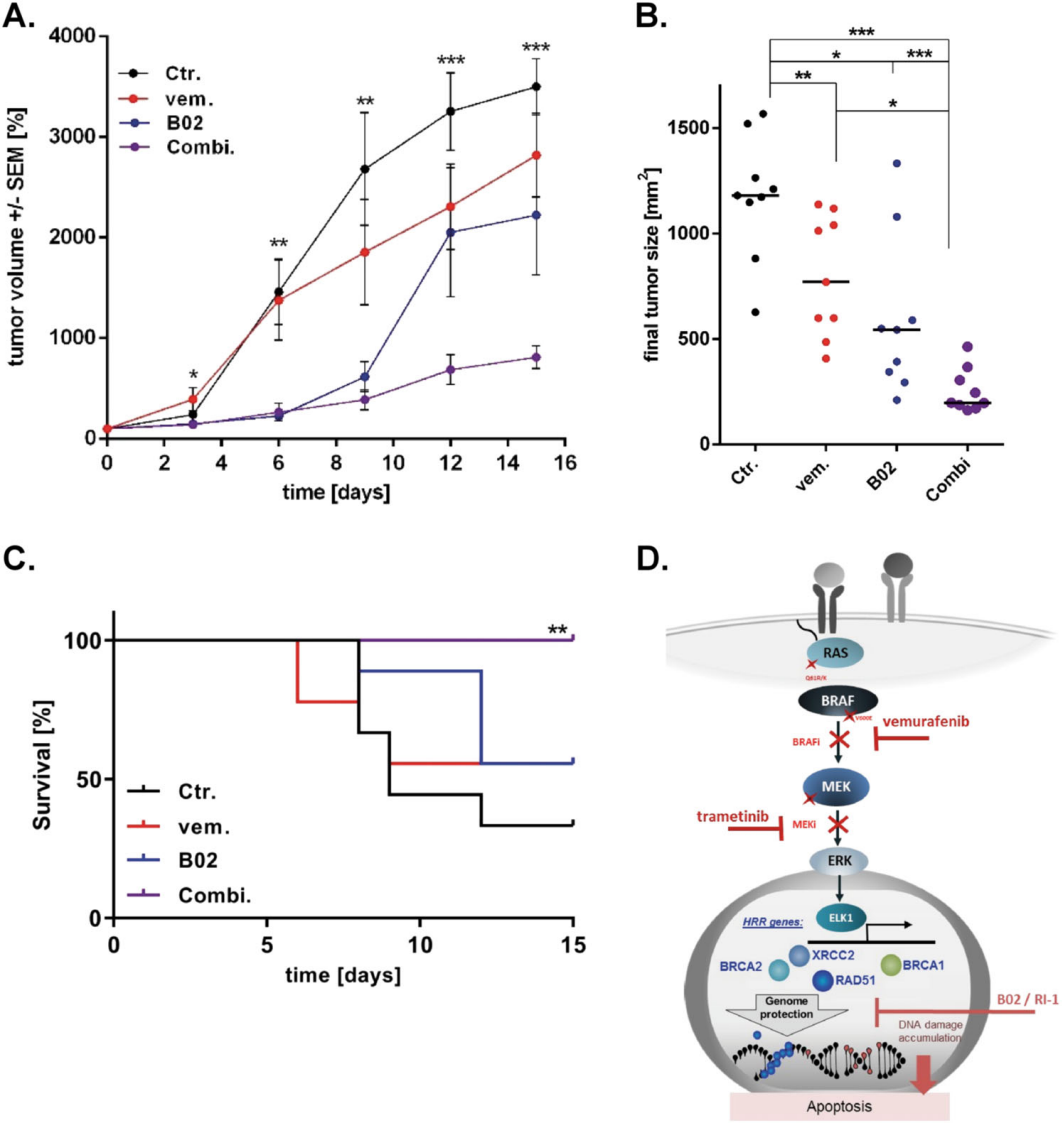
MAPKi resistant melanomas can be effectively treated with a combination of MAPKi and Rad51i in vivo. **a** The xenograft tumor volume in relation to the tumor volume at the start of treatment is indicated. Multiple *t*-test was used to compare the combi. and Ctr. groups. **b** The size of the xenograft tumor of individual mice at the end of the experiment or in sacrificed mice is displayed (mean ± SEM, unpaired Student’s *t*-test). **c** Survival analysis of the xenograft experiment and subsequent Mantel-Cox test. **a–c** All significant differences are marked as asterisks with * for *p* < 0.05, ** for p < 0.01, and *** for *p* < 0.001. **d** Concept of combined MAPK signal inhibition by BRAFi and MEKi and additional Rad51 inhibition as a therapeutic strategy for melanoma treatment.

Finally, based on the promising in vitro data in 2D and 3D models, we analyzed the efficacy of a combined MAPK pathway and Rad51 inhibition in an in vivo mouse model. We found that combined treatment of mice with A375 R xenograft melanoma significantly inhibits tumor growth, while BRAFi or Rad51i alone reduced melanoma tumor growth to a lesser extent (Fig. 5a). This correlated with the effect on the expression of Rad51, ELK1, pERK1/2 and Ki67 in mouse tumors (Supplementary Fig. 5A, B). The final tumor size in the combination therapy mouse group was significantly reduced compared to tumors in the solvent-treated control mouse group and tumors in single-inhibitor treated mice (Fig. 5b). We have also confirmed that the treatments have no effect on the body weight of the mice (Supplementary Fig. 5C). Survival analysis confirmed the benefit of co-inhibitor treatment compared to survival of mice in the other groups (Fig. 5c).

## Discussion

In this work we show that metastatic melanoma cells overexpress genes involved in HRR. In particular, *RAD51* mRNA and protein expression is upregulated in metastatic melanoma cell lines and patient samples and this high expression correlates with a reduced overall survival of melanoma patients. We show that the expression of *RAD51* and other HRR genes is regulated by the MAPK-Elk1 signaling axis in melanoma cells. Furthermore, we demonstrate that an inhibition of Rad51 increases the accumulation of DNA double-strand breaks. Therefore, melanoma cells are sensitive to Rad51 inhibition, which results in an induction of apoptosis. Interestingly, MAPKi resistant cells retain their susceptibility to Rad51 inhibition. Rad51 inhibition enhances the effect of MAPKi treatment in R and RR melanoma cells in vivo and in vitro suggesting that Rad51 is a promising new therapeutic target for the treatment of MAPKi resistant melanoma.

Overexpression of HRR genes, in particular *RAD51* and genes that promote the formation of Rad51 filaments is frequently observed in different cancer cells at both protein and transcript levels^15,35^. Surprisingly, overexpression of these genes is mainly associated with the phenotype of HRR deficiency found in *BRCA1/2* mutant cancer cells^17–19^. Overexpression of *RAD51* is seen as a mechanism to compensate for defects in HRR capacity^19,36,37^. We have shown an overexpression of *RAD51* and other HRR genes, *DDB2*, XRCC2, *BRCA1* and *BRCA2*, in melanoma cell lines and this has a protective role against DNA damage accumulation after genotoxic stress. The increased expression of *RAD51* is connected to aggressiveness and metastatic potential of several cancers including melanoma cells which is associated with reduced patient survival^5,14,38,39^. Here we confirm the specific effect of *RAD51* overexpression on overall survival of melanoma patients and analyzed the underlying mechanism.

Hyperactivation of the MAPK pathway is considered to be the first process during the development of melanoma and is observed in most melanomas^40^. We show that expression of *RAD51* as well as of *XRCC2*, *BRCA1* and *BRCA2* is regulated on a transcriptional level by the MAPK signaling pathway. In search for the downstream transcriptional effectors we focused on cMyc, Stat3 and Elk1. We could show that especially Elk1 is a critical downstream effector mediating increased expression of *Rad51* in melanoma cells. It has been shown in other cancer cells that *RAD51* expression can be transcriptionally regulated by various signaling pathways, including regulation by the MAPK signaling pathway and downstream effectors such as cMyc or EGR and Elk1^20–23^. For example, strong ERK signaling can improve DNA double-strand break repair in glioma cells^24^. In human non-small-cell lung cancer cells, *RAD51* mRNA expression can be induced by gemcitabine-mediated activation of ERK1/2^25^. Regulation of *RAD51* by MAPKi activity is also observed in *KRAS* mutant colon cancer cells. They are dependent on cMyc-induced *RAD51* expression and Rad51-mediated homologous recombination repair^26^. Recently, it has been shown that inhibition of the MAPK pathway in melanoma induces an HRR-defective gene expression signature and that Elk1 and other Ets family members are involved in the regulation of part of the DNA repair genes^22^. Therefore, we assume that Elk1 is one of the critical regulators of *RAD51, XRCC2, BRCA1* and *BRCA2* expression, although regulation by other MAPK pathway effectors cannot be excluded.

In addition to the regulation by the MAPK signal pathway, it is known that the expression of *RAD51, BRCA1* and *BRCA2* is also periodically controlled according to the cycle phases^41^. The expression of these genes reaches a high level in the S or G2 phase, when the sister chromatid is available for efficient HRR. However, we could exclude an effect of MAPKi on the phase distribution of the cell cycle in the applied time period and treatment concentration. Therefore, the data confirm a direct regulatory effect of MAPK signaling activity on the transcription of these genes.

We found that metastatic melanoma cells are susceptible to inhibition of Rad51, while the viability of non-cancerous skin cells was not affected by treatment with Rad51i. These data underline a high dependency of metastatic melanoma cells on Rad51 expression level and activity. Most likely, it is due to the ability of Rad51 overexpressing cells to increase HRR and by this protect DNA in replication fork stalling to cope with an increased level of double-strand DNA breaks^6–8^ as often observed in cancer cells^36,42^. In line with this assumption, we show the induction of DNA damage formation and DNA replication stop as a result of Rad51i treatment.

It is known that cancer cells with defects in a DNA repair pathway develop selective toxicity to genotoxic agents and PARP inhibitors. It is a synthetic lethality of the additional inhibition of the remaining DNA repair mechanisms. For example, a recent study postulates a synthetic lethality of PTEN-deficient melanoma cells to Rad51 inhibition, as PTEN affects non-homologous end joining repair (NHEJ) as an alternative DNA double-strand break repair pathway to HRR^43^. A synthetic lethal relationship is also possible between the NER and the HR mechanism and several mutations and polymorphisms in NER genes are significantly associated with melanoma formation^44,45^. Therefore, the impairment of NER in some of the melanoma cells^46^ may affect sensitivity to Rad51is as well. In addition, Rad51 overexpression occurs frequently in HRR-deficient cells^17,19^. The inhibition of Rad51 and the resulting blockade of the remaining HRR could be sufficient to induce death of melanoma cells and thus explain the high dependence of these cells on Rad51 expression. In addition, melanoma is a cancer with one of the highest average mutation loads^37^. This point might make melanoma cells particularly dependent on the remaining repair mechanism to protect genomic stability and ensure cell survival.

Interestingly, we found that melanoma cells, which developed resistance towards MAPKi are still sensitive to Rad51 inhibition allowing treatment with Rad51is regardless of the resistance status. Most strikingly, we see a synergistic induction of apoptosis as well as reduction of colony formation and melanoma spheroid growth in MAPKi resistant melanoma cells after treatment with Rad51i and MAPKi compared to the treatment effects of the single inhibitors. Furthermore, in most of the in vitro generated R and RR cells there is a remaining effect of MAPKi treatment on cell viability, although it is much lower than in the sensitive original cells. Due to the additionally shown regulation of Rad51 expression by MAPK signaling pathway, we assumed that co-inhibition of MAPK signaling pathway and its downstream effector Rad51 might be effective as two targets of the same pathway against R and RR cells. This co-inhibition was intended to reduce the dose of each inhibitor required for appropriate treatment. It should be noted that in these R and RR cells, we did not observe the pro-proliferative effect of MAPKi treatment described in former publication^47^.

One of the small molecule inhibitors of Rad51 used here, B02 interacts specifically with the ssDNA binding region of Rad51 and thereby reduces the strand exchange^48^, while the second inhibitor, RI-1, irreversibly inhibits polymer formation at the ssDNA^32^. Both inhibitors have already been successfully tested in vitro against some cancer cell types alone or in combination with chemotherapeutic agents^32,49^. B02 has also been tested in xenograft mouse experiments without apparent toxicity and with impressive preclinical activity^31^. Our in vivo approach confirms and complements these data. Rad51 is seen as a promising target in the treatment of various cancers, therefore there are ongoing efforts to discover additional Rad51 inhibitors and use these results in future clinical trials^16,50^. Our data support the application of such inhibitors for the treatment of metastatic melanoma even after the development of resistance to MAPK inhibitors.

## Supplementary information

Supplementary Figure legends-clean copy

Supplementary Figure 1

Supplementary Figure 2

Supplementary Figure 3

Supplementary Figure 4

Supplementary Figure 5

Supplementary Table

## Data Availability

We declare that all the data that support the findings of this study are available from the corresponding author upon request.

## References

[1] Hodis, E. et al. A landscape of driver mutations in melanoma. *Cell* **150**, 251–263 (2012). 22817889 10.1016/j.cell.2012.06.024 PMC3600117

[2] Krauthammer, M. et al. Exome sequencing identifies recurrent somatic RAC1 mutations in melanoma. *Nat. Genet.* **44**, 1006–1014 (2012). 22842228 10.1038/ng.2359 PMC3432702

[3] Wei, Q., Cheng, L., Xie, K., Bucana, C. D. & Dong, Z. Direct correlation between DNA repair capacity and metastatic potential of K-1735 murine melanoma cells. *J. Invest Dermatol.* **108**, 3–6 (1997). 8980277 10.1111/1523-1747.ep12285608

[4] Winnepenninckx, V. et al. Gene expression profiling of primary cutaneous melanoma and clinical outcome. *J. Natl Cancer Inst.* **98**, 472–482 (2006). 16595783 10.1093/jnci/djj103

[5] Kauffmann, A. et al. High expression of DNA repair pathways is associated with metastasis in melanoma patients. *Oncogene* **27**, 565–573 (2008). 17891185 10.1038/sj.onc.1210700

[6] Haaf, T., Golub, E. I., Reddy, G., Radding, C. M. & Ward, D. C. Nuclear foci of mammalian Rad51 recombination protein in somatic cells after DNA damage and its localization in synaptonemal complexes. *Proc. Natl Acad. Sci. USA* **92**, 2298–2302 (1995). 7892263 10.1073/pnas.92.6.2298 PMC42471

[7] Tashiro, S., Walter, J., Shinohara, A., Kamada, N. & Cremer, T. Rad51 accumulation at sites of DNA damage and in postreplicative chromatin. *J. Cell Biol.* **150**, 283–291 (2000). 10908572 10.1083/jcb.150.2.283 PMC2180223

[8] Bhattacharya, S. et al. RAD51 interconnects between DNA replication, DNA repair and immunity. *Nucleic Acids Res.* **45**, 4590–4605 (2017). 28334891 10.1093/nar/gkx126 PMC5416901

[9] Tarsounas, M., Davies, A. A. & West, S. C. RAD51 localization and activation following DNA damage. *Philos. Trans. R. Soc. Lond. B Biol. Sci.* **359**, 87–93 (2004). 15065660 10.1098/rstb.2003.1368 PMC1693300

[10] Baumann, P. & West, S. C. Role of the human RAD51 protein in homologous recombination and double-stranded-break repair. *Trends Biochem. Sci.* **23**, 247–251 (1998). 9697414 10.1016/s0968-0004(98)01232-8

[11] Krejci, L., Altmannova, V., Spirek, M. & Zhao, X. Homologous recombination and its regulation. *Nucleic Acids Res.* **40**, 5795–5818 (2012). 22467216 10.1093/nar/gks270 PMC3401455

[12] Maacke, H. et al. Over-expression of wild-type Rad51 correlates with histological grading of invasive ductal breast cancer. *Int. J. Cancer* **88**, 907–913 (2000). 11093813 10.1002/1097-0215(20001215)88:6<907::aid-ijc11>3.0.co;2-4

[13] Takenaka, T. et al. Combined evaluation of Rad51 and ERCC1 expressions for sensitivity to platinum agents in non-small cell lung cancer. *Int. J. Cancer* **121**, 895–900 (2007). 17417781 10.1002/ijc.22738

[14] Mitra, A. et al. Overexpression of RAD51 occurs in aggressive prostatic cancer. *Histopathology* **55**, 696–704 (2009). 20002770 10.1111/j.1365-2559.2009.03448.x PMC2856636

[15] Raderschall, E. et al. Elevated levels of Rad51 recombination protein in tumor cells. *Cancer Res.* **62**, 219–225 (2002). 11782381

[16] Ward, A., Khanna, K. K. & Wiegmans, A. P. Targeting homologous recombination, new pre-clinical and clinical therapeutic combinations inhibiting RAD51. *Cancer Treat. Rev.* **41**, 35–45 (2015). 25467108 10.1016/j.ctrv.2014.10.006

[17] Klein, H. L. The consequences of Rad51 overexpression for normal and tumor cells. *DNA Repair (Amst.)* **7**, 686–693 (2008). 18243065 10.1016/j.dnarep.2007.12.008 PMC2430071

[18] Nagathihalli, N. S. & Nagaraju, G. RAD51 as a potential biomarker and therapeutic target for pancreatic cancer. *Biochim. Biophys. Acta* **1816**, 209–218 (2011). 21807066 10.1016/j.bbcan.2011.07.004

[19] Martin, R. W. et al. RAD51 up-regulation bypasses BRCA1 function and is a common feature of BRCA1-deficient breast tumors. *Cancer Res.* **67**, 9658–9665 (2007). 17942895 10.1158/0008-5472.CAN-07-0290

[20] Hine, C. M. et al. Regulation of Rad51 promoter. *Cell Cycle* **13**, 2038–2045 (2014). 24781030 10.4161/cc.29016 PMC4111695

[21] Luoto, K. R. et al. Tumor cell kill by c-MYC depletion: role of MYC-regulated genes that control DNA double-strand break repair. *Cancer Res.* **70**, 8748–8759 (2010). 20940401 10.1158/0008-5472.CAN-10-0944

[22] Maertens, O. et al. MAPK pathway suppression unmasks latent DNA repair defects and confers a chemical synthetic vulnerability in BRAF-, NRAS-, and NF1-mutant melanomas. *Cancer Discov.* **9**, 526–545 (2019). 30709805 10.1158/2159-8290.CD-18-0879 PMC10151004

[23] Estrada-Bernal, A. et al. MEK inhibitor GSK1120212-mediated radiosensitization of pancreatic cancer cells involves inhibition of DNA double-strand break repair pathways. *Cell Cycle* **14**, 3713–3724 (2015). 26505547 10.1080/15384101.2015.1104437 PMC4825728

[24] Golding, S. E. et al. Pro-survival AKT and ERK signaling from EGFR and mutant EGFRvIII enhances DNA double-strand break repair in human glioma cells. *Cancer Biol. Ther.* **8**, 730–738 (2009). 19252415 10.4161/cbt.8.8.7927 PMC2863288

[25] Tsai, M. S., Kuo, Y. H., Chiu, Y. F., Su, Y. C. & Lin, Y. W. Down-regulation of Rad51 expression overcomes drug resistance to gemcitabine in human non-small-cell lung cancer cells. *J. Pharmacol. Exp. Therapeutics* **335**, 830–840 (2010). 10.1124/jpet.110.173146 20855443

[26] Kalimutho, M. et al. Enhanced dependency of KRAS-mutant colorectal cancer cells on RAD51-dependent homologous recombination repair identified from genetic interactions in Saccharomyces cerevisiae. *Mol. Oncol.* **11**, 470–490 (2017). 28173629 10.1002/1878-0261.12040 PMC5527460

[27] Sun, J., Carr, M. J. & Khushalani, N. I. Principles of targeted therapy for melanoma. *Surg. Clin. North Am.* **100**, 175–188 (2020). 31753111 10.1016/j.suc.2019.09.013

[28] Makino, E. et al. Melanoma cells resistant towards MAPK inhibitors exhibit reduced TAp73 expression mediating enhanced sensitivity to platinum-based drugs. *Cell Death Dis.* **9**, 930 (2018). 30206212 10.1038/s41419-018-0952-8 PMC6133963

[29] Meier, F. et al. Human melanoma progression in skin reconstructs: biological significance of bFGF. *Am. J. Pathol.* **156**, 193–200 (2000). 10623667 10.1016/S0002-9440(10)64719-0 PMC1868639

[30] Sinnberg, T. et al. A nexus consisting of beta-catenin and Stat3 attenuates BRAF inhibitor efficacy and mediates acquired resistance to Vemurafenib. *EBioMedicine* **8**, 132–149 (2016). 27428425 10.1016/j.ebiom.2016.04.037 PMC4919613

[31] Huang, F. & Mazin, A. V. A small molecule inhibitor of human RAD51 potentiates breast cancer cell killing by therapeutic agents in mouse xenografts. *PLoS One* **9**, e100993 (2014). 24971740 10.1371/journal.pone.0100993 PMC4074124

[32] Budke, B. et al. RI-1: a chemical inhibitor of RAD51 that disrupts homologous recombination in human cells. *Nucleic Acids Res.* **40**, 7347–7357 (2012). 22573178 10.1093/nar/gks353 PMC3424541

[33] Pierce, A. J., Johnson, R. D., Thompson, L. H. & Jasin, M. XRCC3 promotes homology-directed repair of DNA damage in mammalian cells. *Genes Dev.* **13**, 2633–2638 (1999). 10541549 10.1101/gad.13.20.2633 PMC317094

[34] Cancer Genome Atlas, N. Genomic classification of cutaneous melanoma. *Cell* **161**, 1681–1696 (2015). 26091043 10.1016/j.cell.2015.05.044 PMC4580370

[35] Pitroda, S. P. et al. DNA repair pathway gene expression score correlates with repair proficiency and tumor sensitivity to chemotherapy. *Sci. Transl. Med.* **6**, 229ra242 (2014). 10.1126/scitranslmed.3008291 PMC4889008 24670686

[36] Schild, D. & Wiese, C. Overexpression of RAD51 suppresses recombination defects: a possible mechanism to reverse genomic instability. *Nucleic Acids Res.* **38**, 1061–1070 (2010). 19942681 10.1093/nar/gkp1063 PMC2831301

[37] Chalmers, Z. R. et al. Analysis of 100,000 human cancer genomes reveals the landscape of tumor mutational burden. *Genome Med.* **9**, 34 (2017). 28420421 10.1186/s13073-017-0424-2 PMC5395719

[38] Sarasin, A. & Dessen, P. DNA repair pathways and human metastatic malignant melanoma. *Curr. Mol. Med.* **10**, 413–418 (2010). 20455851 10.2174/156652410791317011

[39] Jewell, R. et al. Patterns of expression of DNA repair genes and relapse from melanoma. *Clin. Cancer Res.* **16**, 5211–5221 (2010). 20705614 10.1158/1078-0432.CCR-10-1521 PMC2972254

[40] Shtivelman, E. et al. Pathways and therapeutic targets in melanoma. *Oncotarget* **5**, 1701–1752 (2014). 24743024 10.18632/oncotarget.1892 PMC4039128

[41] Whitfield, M. L. et al. Identification of genes periodically expressed in the human cell cycle and their expression in tumors. *Mol. Biol. Cell* **13**, 1977–2000 (2002). 12058064 10.1091/mbc.02-02-0030. PMC117619

[42] Kolinjivadi, A. M. et al. Moonlighting at replication forks—a new life for homologous recombination proteins BRCA1, BRCA2 and RAD51. *FEBS Lett.* **591**, 1083–1100 (2017). 28079255 10.1002/1873-3468.12556

[43] Turchick, A., Liu, Y., Zhao, W., Cohen, I. & Glazer, P. M. Synthetic lethality of a cell-penetrating anti-RAD51 antibody in PTEN-deficient melanoma and glioma cells. *Oncotarget* **10**, 1272–1283 (2019). 30863489 10.18632/oncotarget.26654 PMC6407680

[44] Millikan, R. C. et al. Polymorphisms in nucleotide excision repair genes and risk of multiple primary melanoma: the Genes Environment and Melanoma Study. *Carcinogenesis* **27**, 610–618 (2006). 16258177 10.1093/carcin/bgi252

[45] Povey, J. E. et al. DNA repair gene polymorphisms and genetic predisposition to cutaneous melanoma. *Carcinogenesis* **28**, 1087–1093 (2007). 17210993 10.1093/carcin/bgl257

[46] Belanger, F., Rajotte, V. & Drobetsky, E. A. A majority of human melanoma cell lines exhibits an S phase-specific defect in excision of UV-induced DNA photoproducts. *PloS ONE* **9**, e85294 (2014). 24416382 10.1371/journal.pone.0085294 PMC3885708

[47] Moriceau, G. et al. Tunable-combinatorial mechanisms of acquired resistance limit the efficacy of BRAF/MEK cotargeting but result in melanoma drug addiction. *Cancer Cell* **27**, 240–256 (2015). 25600339 10.1016/j.ccell.2014.11.018 PMC4326539

[48] Huang, F. et al. Identification of specific inhibitors of human RAD51 recombinase using high-throughput screening. *ACS Chem. Biol.* **6**, 628–635 (2011). 21428443 10.1021/cb100428c PMC3117970

[49] Huang, F., Mazina, O. M., Zentner, I. J., Cocklin, S. & Mazin, A. V. Inhibition of homologous recombination in human cells by targeting RAD51 recombinase. *J. Med Chem.* **55**, 3011–3020 (2012). 22380680 10.1021/jm201173g

[50] Hengel, S. R., Spies, M. A. & Spies, M. Small-molecule inhibitors targeting DNA repair and DNA repair deficiency in research and cancer therapy. *Cell Chem. Biol.* **24**, 1101–1119 (2017). 28938088 10.1016/j.chembiol.2017.08.027 PMC5679738

