# Supplementary material for: Targeting Rad51 as a strategy for the treatment of melanoma cells resistant to MAPK pathway inhibition
Source: Cell Death Dis. 2020 Jul 2;11(7):581. doi: 10.1038/s41419-020-2702-y (PMC7385107; doi:10.1038/s41419-020-2702-y)
Supplement: Supplementary file 1 — Supplementary Figure legends-clean copy [file 41419_2020_2702_MOESM1_ESM.docx]

**Supplementary Figure legends**

**Suppl. Figure 1: A.** Overall survival of melanoma patients depending on HRR gene expression (high expression: blue line, n=174, low expression: red line, n=201). Cutaneous melanoma (SKCM) patient data from the TCGA database were used. **B**. BRAF mutation status (wt: wild type or (V600) mutated), previous systemic therapy and the RAD51 level detected in the immuno-histological staining (described and shown in Figure 1D) are listed for 25 analyzed metastatic melanoma samples (MM1-MM25).

**Suppl. Figure 2: A.** Genes that are considered significantly downregulated in the analysis of Figure 2 A. are listed (p≤0.05; fold upregulation: > 2.5; fold reduction (-1/(fold change)) < -2.5 ). HRR: homologous recombination repair, MMR: mismatch repair pathway, BER: base excision repair pathway, NER: nucleotide excision repair pathway. **B**. The cell cycle phase distribution of different melanoma cell lines after vemurafenib treatment (vem., 2 or 5µM, 24h or 48h) or DMSO treatment (Ctr., 0.02%) is displayed (mean value of triplicates +/- SD). **C.** The binding sites of the critical transcription factors in the indicated HRR gene promoter regions were determined via the ENCODE website (b.r.: binding region). **D.** Immunoblot analysis using Stat3 and cMyc knockdown samples (siRNA transfection, siStat3, sicMyc) of the A375 cells (48h after transfection). Samples of non-coding siRNA transfected cells were used as control (siCtr.). The Rad51 protein level in the respective samples were quantified and normalized to the correspondent actin level (lower graph, n=3, mean +/- SD, Unpaired Student’s t-test, ns: not significant: p>/=0.05). **E.** Immunoblot analysis was performed using Stat3 overexpressing (Stat3OE) cells of the SK-MEL19 cell line and respective control cells (Ctr.). **F** RAD51 Promotor Reporter Assay with or without co-transfected siRNAs against Elk1 (siCtr, siELk1A or siElk1B). The luciferase signal was detected 72h after transfection and normalized to secreted alkaline phosphatase activity. The cells were treated with 2µM vemurafenib (vem.) or 20nM trametinib (tra.) for 48h (an exemplary experiment, mean values +/- SD). **G.** ChIP analysis to detect Elk1 binding to the RAD51 promoter region. The pull-down was performed with antibodies against Elk1 (αElk1 Ab) and against Histone H3 (αHiston H3Ab) as positive control (an exemplary experiment, mean values +/- SD).

**Suppl. Figure 3: A.** S, R and RR melanoma cell lines were treated with different concentrations of vemurafenib (vem.) or additionally with trametinib (tra.) for 3 days. Subsequent viability analyses were performed to confirm treatment resistance in R and RR cells. **B.** Immunoblot analysis for Rad51 was performed using indicated S, R and RR cells. Actin served as loading control **C.** The reduction in HRR capacity was confirmed by the pDRGFP reporter assay after SceI endonuclease expression and B02 and RI-1 treatment. Exemplary data of a FACS experiment is presented (left graph). The percentage of HRR-efficient cells (GFP-positive population) is given (right graph, mean value of triplicates +/- SD). **D.** The effect of B02 treatment (15µM, 48h) on DNA replication was analyzed in S and R cells by BrdU incorporation Assay. The data of an exemplary experiment are presented (left graph). The percentage of BrdU positive population is displayed (right graph, mean value of triplicates +/- SD).

**Suppl. Figure 4: A.** Immunoblot analysis of A375 S and R cells treated with vemurafenib (vem., 5 or 10µM) for 24h. Nuclear (nuc.) and cytoplasmic (cyt.) parts of the lysates are analyzed. **B.** Immunoblot analysis of A375 S and R cells treated with vemurafenib (vem., 5µM), B02 (10µM.) or with the combination of these inhibitors for 24h. **C.** Immunofluorescence staining for Rad51 in A375 R cells treated with vemurafenib (vem., 5μM), B02 (10µM) or the combination of these inhibitors for 48h. The quantified fluorescence intensity of Rad51 is indicated as Rad51 expression relative to the level in the untreated Control cells (Ctr.). **D**. Immunofluorescence staining for pH2AX (AlexaFluor647, red) and nuclei (Yo-Pro (green). SK-MEL28RR cells were treated for 48h with vemurafenib (vem., 5μM), trametinib (tra., 50nM), B02 (10µM) or the combination (Combi.) of these inhibitors (left graph; scale bars: 20 µm. The pH2AX foci number per cell was quantified for each treatment group (right graph). **E.** Cell cycle analysis was performed after treatment with vemurafenib (vem., 5µM), B02 (10µM) or a combination of the inhibitors for 5 days (PI staining). DMSO-treated cells (0.02%) served as control (Ctr.) (mean of triplicates ± SEM).**F.** Colony formation is analyzed 7 days after treatment with vemurafenib (vem., 5µM), additional treatment with trametinib (tra., 50nM), RI-1 (10µM) or the indicated combination of these inhibitors. Exemplary images of the colonies can be seen. **G.** Melanoma spheroids were treated with DMSO (Ctr., 0.02%), vemurafenib (vem. 5µM), trametinib (tra. 100nM), RI-1 (20µM), B02 (10 µM) or a combination of these inhibitors in collagen for 5 days. **H.** Two different siRNAs against Rad51 (siRad51A and B) or non-coding Control siRNA (siCtr.) were transfected into melanoma cells. Viability analysis was performed after treatment with different concentrations of vemurafenib for 5 days (mean values of quintuplicates +/- SD).

**Suppl. Figure 5: A.** Representative immunohistochemical pictures of A375 R xenograft melanomas (100x magnification). Formalin-fixed and paraffine embedded tumors were used for staining of Rad51, phospho-ERK1/2, ELK1 and the proliferation marker Ki67 (red signal). **B.** Staining intensities were semi-quantified using a score of 1-3 (weak, moderate, strong staining) and counting the percentage of the corresponding cell populations (X% x 3 + Y% x2 + Z% x 1 = Quick Score) or calculating the percentage of positive cells (only for Ki67). Scale bars: 200 µm. **C.** The body weight of the mice in each treatment group during the xenograft experiment shown in Figure 5 is shown (9 mice per group, mean +/- SD)
