# Supplementary figures and images for: Targeting Rad51 as a strategy for the treatment of melanoma cells resistant to MAPK pathway inhibition
Source: Cell Death Dis. 2020 Jul 2;11(7):581. doi: 10.1038/s41419-020-2702-y (PMC7385107; doi:10.1038/s41419-020-2702-y)

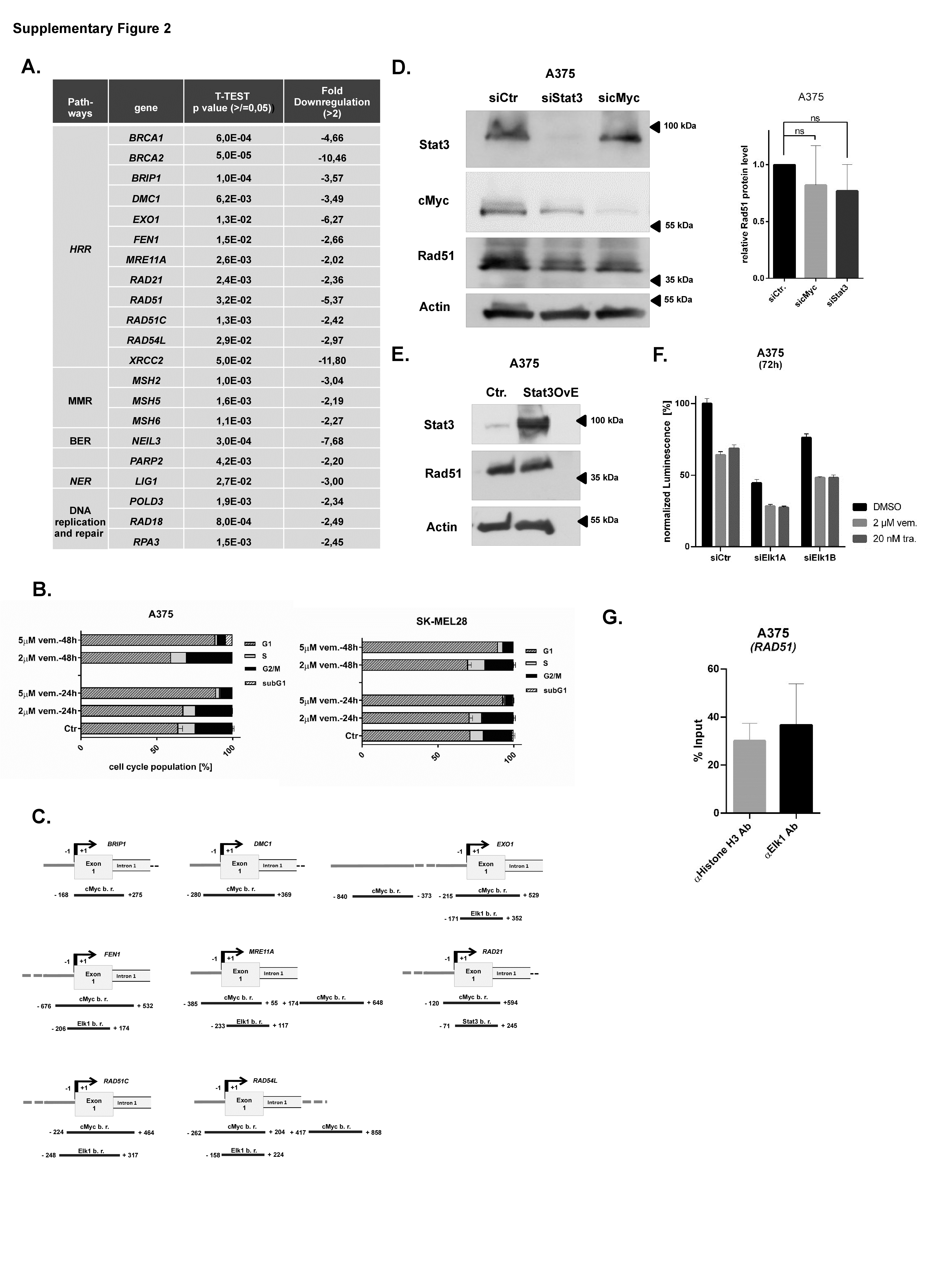

Supplement: Supplementary file 3 — Supplementary Figure 2 [file 41419_2020_2702_MOESM3_ESM.tif]

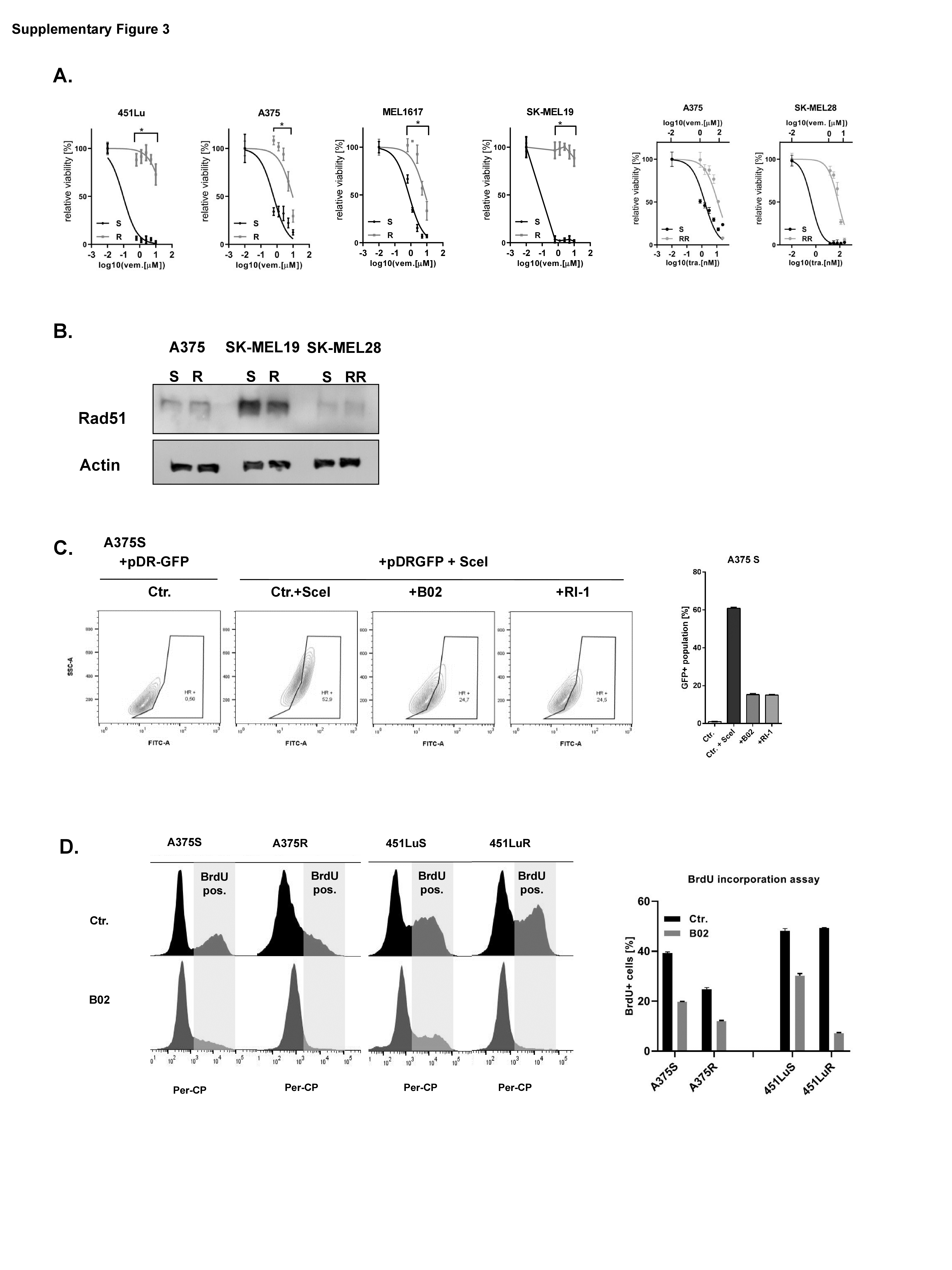

Supplement: Supplementary file 4 — Supplementary Figure 3 [file 41419_2020_2702_MOESM4_ESM.tif]

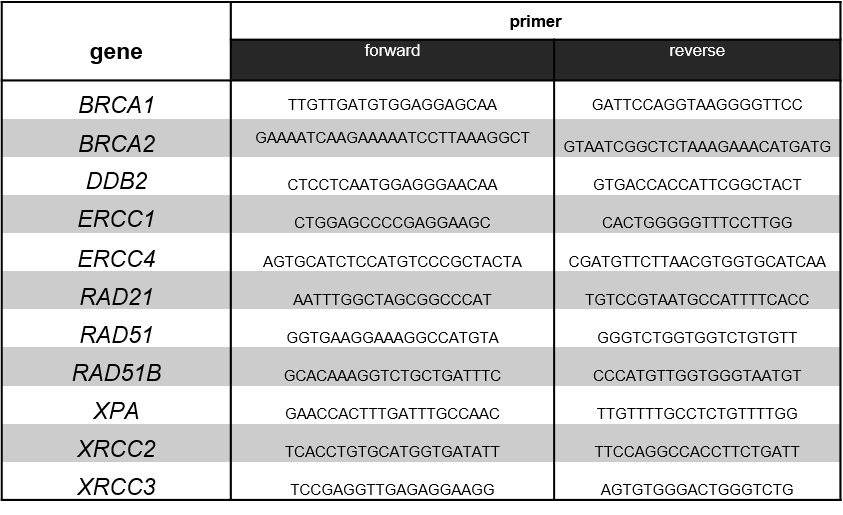


**Supplementary Table 1**

Supplement: Supplementary file 7 — Supplementary Table [file 41419_2020_2702_MOESM7_ESM.docx]
